# To improve one's own self: how and when perceived leader competence promotes employees' self improvement feedback-seeking behavior

**DOI:** 10.3389/fpsyg.2025.1674270

**Published:** 2026-03-02

**Authors:** Xiaokun Liu, Boyang Gong, Qing Zheng, Shuai Wang, Siyuan Liu, Wei Zhang

**Affiliations:** 1 State Key Laboratory of Nuclear Power Safety Technology and Equipment, China Nuclear Power Engineering Co., Ltd., Shenzhen, China; 2 School of Government, Beijing Normal University, Beijing, China; 3 Institute of Developmental Psychology, Beijing Normal University, Beijing, China; 4 School of Management Science and Engineering, University of Jinan, Jinan, China

**Keywords:** perceived leader competence, hope, LMX, feedback-seeking behavior, self-improvement perspective

## Abstract

**Introduction:**

The extant literature has stressed the importance of employees' feedback-seeking behavior. In this paper, we focus on the self-improvement aspect of this behavior and its activator, drawing on the self-improvement motives perspective to construct a theoretical model in which perceived leader competence predicts employees' self-improvement feedback-seeking via the mediating role of employee hope.

**Methods:**

A three-wave study was conducted with 660 employees from a Chinese company to test our hypotheses.

**Results:**

Results showed that employee hope partially mediated the relationship between perceived leader competence and self-improvement feedback-seeking, with the indirect effect being marginally significant at average levels of LMX and significantly positive at high levels of LMX. Additionally, LMX moderated both the relationship between perceived leader competence and employee hope and the relationship between employee hope and self-improvement feedback-seeking, such that these relationships were stronger when LMX was high.

**Discussion:**

Theoretical and practical implications of these findings, as well as directions for future research, are discussed.

## 1 Introduction

Due to external pressure or internal motives, employees have the desire to improve their performance or themselves in the workplace (Cheng et al., 2024). One important way to achieve improvement is through seeking feedback (especially self-improvement feedback) from others, which means obtaining “information (i.e., feedback information) that is useful for improving personal attributes such as knowledge, skills and abilities” (Janssen and Prins, 2007, p. 237). Prior research has indicated that leader behaviors or leadership styles can promote employees' feedback-seeking behavior. For instance, supervisor support increases the frequency of employees' feedback-seeking (Chen et al., 2021; Nifadkar et al., 2012); leadership styles such as ethical leadership, transformational leadership, inclusive leadership, and leader humility are positively related to employees' feedback-seeking behavior (Moss et al., 2020; Pan and Lin, 2015; Qian et al., 2022; Song et al., 2023).

Although the extant literature has increased our understanding of facilitators of employees' feedback-seeking and how leader factors can enhance subordinates' feedback-seeking behavior, our knowledge of the improvement nature of feedback-seeking and its potential leader antecedents is incomplete in two ways. First, most of the extant research does not indicate the self-improvement side of feedback-seeking. Thus, while researchers have developed theoretical models to explore the antecedents of feedback-seeking behavior, they have focused mainly on feedback about one's overall performance, the technical aspects of the firm, role expectations and so on VandeWalle et al. (2000). We know less about the case in which employees intend to obtain feedback aimed at self-improvement. Studying this side of feedback-seeking is important because feedback toward self-improvement plays a great role in performance advancement (Anseel et al., 2007).

Second, leaders play crucial roles in motivating subordinates' feedback-seeking behavior. Prior research has stressed either the simulative or sometimes impeditive functions of leader-specific behaviors or leadership styles (Ashford et al., 2016). However, subordinates' perceptions of leader competence have mostly been neglected. Indeed, leader competence can lead to positive work results for subordinates, such as increasing their knowledge sharing and helping them achieve performance improvement (Levenson et al., 2006; Swanson et al., 2020). Moreover, from a capacity-building perspective, leader competence can be seen as a micro-foundation of organizational capacity building, which channels employees toward self-improvement behaviors such as feedback-seeking, ultimately enhancing performance (Saputra et al., 2024). Based on this, we target an important question that has yet to be fully addressed: Will the perception of leaders' competence influence employees' self-improvement feedback-seeking? If so, how does this happen?

To solve these problems, we applied the self-motives perspective (here, we mainly focused on self-improvement motives) to indicate how and when perceived leader competence lead employees to engage in self-improvement feedback-seeking behaviors (Anseel et al., 2007). The self-improvement motivation perspective contends that people are motivated to improve their traits, abilities and skills, and it emphasizes the genuine improvement of individuals (Anseel et al., 2007; Sedikides and Strube, 1997). In regard to the activator of employees' self-improvement motivation, leader competency counts (Levenson et al., 2006). This is because employees are more motivated and willing to accept the influence of leaders who are more competent (Byun et al., 2017; Wei et al., 2018).

In addition, we argue that employee hope (i.e., the perceived capability to derive pathways to desired goals and motivate oneself via agency thinking to use those pathways, Snyder, 2002, p. 249) can explain the self-improvement process between perceived leader competence and employees' feedback-seeking. This is because hope contains both the will (i.e., want to do) and way (i.e., can do) paths toward self-improvement (Snyder et al., 1996). In other words, perceived leader competence has the potential to increase employees' willingness and confidence (i.e., high-level hope) to improve themselves, which in turn allows employees to engage in self-improvement behaviors such as seeking self-improvement feedback.

We further explore the moderating factors that influence the aforementioned paths. Research on self-motives indicates that social context, such as relationships with others, can influence the self-improvement process (Sedikides and Strube, 1997). Because we focused on supervisor-subordinate interactions in the current study, we propose that LMX, that is, the relationship quality among employees and their leaders (Dulebohn et al., 2012), will play a moderating role. Specifically, we argue that employees will be more motivated (i.e., sense high-level hope) by their competent leaders, and will transfer self-improvement motivation (i.e., hope) more to feedback-seeking behavior when they have good relationships with their leader (i.e., high-level LMX). This is because high levels of LMX indicate mutual trust, respect, and obligations between subordinates and their leaders (Matta et al., 2015), which may have the potential to enhance the functions of perceived leader competence and employee hope.

**Figure 1:**
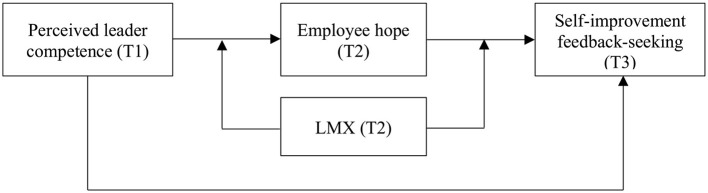
Theoretical model.

Our research makes three contributions. First, we contribute to the feedback-seeking literature by demonstrating how self-improvement motives play a role in the feedback-seeking process and identifying one potential activator (i.e., perceived leader competence) of employees' self-improvement feedback-seeking (Anseel et al., 2007). Second, we contribute to the literature by connecting an important motivational state (i.e., hope) with studies on employees (Luthans and Jensen, 2002). Finally, we contribute to LMX theory by distinguishing the moderating effects of LMX in different contexts (i.e., leader competence, employee motivational state, and employee self-improvement behavior). Figure 1 presents a summary of the theoretical model.

## 2 Theory and hypotheses

### 2.1 Perceived leader competence and employee self-improvement feedback-seeking

Self-improvement feedback-seeking is defined as “seeking (feedback) information that is useful for improving personal attributes such as knowledge, skills and abilities” (Janssen and Prins, 2007, p. 237). While not focusing on the self-improvement side, prior research has broadly uncovered three categories of antecedents of feedback-seeking behavior, inclusive of *seeker antecedents* such as emotional competence and demographic factors (Finkelstein et al., 2003; Kim et al., 2009); *target antecedents* such as target trustworthiness and credibility (Anseel et al., 2015; Choi et al., 2014); and *context antecedents* such as uncertainty and cultural factors (Anseel and Lievens, 2007; MacDonald et al., 2013). Because perceived leader competence portends *target antecedents* (i.e., the feedback targets are trusted and reliable; Ashford et al., 2016), it may increase employees' self-improvement feedback-seeking.

Specifically, when employees believe that their leaders are competent, they tend to believe that their leaders can give them high-quality guidance, help them conquer performance obstacles, and eventually can provide them with accurate and useful feedback (House, 1971; Kong et al., 2023). In other words, competent leaders can satisfy employees' requests for self-improvement feedback. As such, employees are inclined to obtain feedback for improvement from their competent leaders. Accordingly, we hypothesize the following:

**H1:** Perceived leader competence is positively related to employees' self-improvement feedback-seeking.

### 2.2 The mediating role of employee hope

Hope refers to a positive motivational state characterized by a sense of successful *agency* (i.e., goal-directed determination) and *pathways* (i.e., planning of ways to meet goals; Snyder, 2002). Research has indicated that hope can be triggered by positive stimuli such as positive emotions (Ouweneel et al., 2012) and an individual's growth mindset (Lin et al., 2018). While leader competence serves as a positive stimulus that can be helpful for employees' goal progress, performance, learning, and development (Kong et al., 2023), we argue that perceived leader competence may lead to high levels of employee hope.

On the one hand, competent leaders are able to provide employees with accurate evaluations and equip them with matched resources such as useful feedback (Antonakis and House, 2014; Kong et al., 2023), which can help employees create paths toward their goals. In this vein, perceived high-competence leaders will increase the *pathway* component of hope (i.e., ways to achieve desired goals). On the other hand, when guided by a competent leader, employees will be more motivated to take part in their work and show a higher level of followership. In other words, competent leaders can act as activators of employees' willingness to achieve goals, ultimately acting on the *agency* competent of hope (i.e., willingness and determination to achieve desired goals). Accordingly, we hypothesize the following:

**H2:** Perceived leader competence is positively related to employee hope.

Self-improvement feedback-seeking refers to the behavior that individuals use to obtain feedback information to improve themselves (Janssen and Prins, 2007). It is actually an individual's proactive behavior (Grant and Ashford, 2008) and is defined as a self-initiated and future-oriented action that aims to change and improve the situation or oneself (Crant, 2000). According to the proactive motivation model, “*can do*” and “*reason to*” are crucial motivations for individuals' proactivity (Parker et al., 2010). Because hope contains *pathways* and *agency* components that correspond to “*can do*” and “*reason to*” motivation, respectively (Parker et al., 2010; Snyder et al., 1991), we argue that employee hope will increase employees' self-improvement feedback-seeking. More specifically, when employees have high levels of hope, they not only have the ability to work out ways to achieve goals (i.e., can do motivation) but are also willing to improve themselves (i.e., reason for motivation; Snyder, 2002; Snyder et al., 1991). As such, high-level hope guarantees the ways and will of employees to perform proactive behavior, including self-improvement feedback-seeking (Snyder et al., 1991). To support our argument indirectly, research has indicated that hope, as a personal resource, can lead to employees' engagement at their work (Ouweneel et al., 2012).

Combined with Hypothesis 2, which predicts the relationship between perceived leader competence and employee hope, we further propose that hope plays a mediating role in the relationship between perceived leader competence and employees' self-improvement feedback-seeking. Accordingly, we hypothesize the following:

**H3:** Employee hope mediates the relationship between perceived leader competence and self-improvement feedback-seeking.

### 2.3 The moderating role of LMX

In regard to leader-subordinate interactions (including feedback processes) at work, the relationship quality between them plays a vital role (Lam et al., 2017; Lan et al., 2020; Matta et al., 2015; Zhu et al., 2023). For instance, research has indicated that employees interpret the same leader behavior differently when they form different relationships with leaders (Xing et al., 2021; Zhu et al., 2023). As such, leader-subordinate relationship quality may also alter employees' perceptions of leaders' competence and its subsequent impacts, as well as employees' motivational states related to their behaviors. Accordingly, we propose that LMX, which is the relationship quality between subordinates and their leaders (Dulebohn et al., 2012), moderates the relationships between perceived leader competence, employee hope, and self-improvement feedback-seeking relationships.

First, we argue that the effect of perceived leader competence on hope varies with LMX quality. In a high LMX context, employees can gain more attention and support from leaders and can acquire resources at need (Xiong et al., 2021; Zhu et al., 2023). Because employees are equipped with *pathways* and *agency* from their competent leaders, the extra attention, support, and resources derived from high-quality LMX will cultivate the benefits of perceived leader competence for employees. In contrast, when LMX quality is low, employees tend to obtain less support and resources from leaders, which will discount the effect of their perceptions on leader competence. As such, perceived leader competence will have a stronger effect on employee hope in a high-than in a low-LMX context.

Second, we argue that LMX quality moderates the relationship between employee hope and self-improvement feedback-seeking from leaders. LMX research contends that employees with high-quality LMX are less afraid of failure and are more likely to be given greater autonomy in their own tasks (Martinaityte and Sacramento, 2013). Because fearlessness of failure and enough autonomy are important conditions that transfer employees' positive emotional state (i.e., hope) to proactive behaviors (Bauer et al., 2007; Den Hartog and Belschak, 2012; Parker et al., 2006), employees' hope will have a stronger positive relationship with employee self-improvement feedback-seeking in a high-LMX context. In other words, low-level LMX cannot increase employees' security of failure and autonomy for work arrangement, and the relationship between hope and employee self-improvement feedback-seeking will not be strengthened in this context. To support this indirectly, research has indicated that high-quality LMX helps employees transfer their creativity to better performance (Martinaityte and Sacramento, 2013). Most importantly, prior research suggests that competent and agile leaders foster employee hope and promote learning-oriented feedback seeking, particularly in uncertain environments (Syamsir et al., 2025). Accordingly, we hypothesize the following:

**H4:** LMX moderates the relationship between (a) perceived leader competence and employee hope, and (b) employee hope and self-improvement feedback-seeking such that these relationships will be stronger when LMX is higher than when LMX is lower.

## 3 Method

### 3.1 Sample and procedures

We recruited 755 full-time employees from a Chinese nuclear power company to participate in our study. To reduce potential common method bias (Podsakoff et al., 2012), a three-wave design was employed to collect our research data. The time interval between each wave was approximately 1 month. Specifically, in the Time 1 survey, employees were asked to provide their demographic information and rate the competence of their immediate leaders. In the Time 2 survey, employees were asked to rate their hope for themselves and their LMX with their immediate leaders. In the time 3 survey, employees were asked to rate their self-improvement feedback-seeking behavior from leaders.

After matching the questionnaires, our final sample contained 660 valid participants who completed all three waves of our surveys (response rate across all three waves = 85.16%). A series of independent samples T-tests on demographic variables (i.e., gender, age, education level, and tenure) between T1 and T3 revealed no significant differences. Owing to the nature of the nuclear company, 91.4% of them were male. Their average age was 35.91 years, with an average tenure of 13.07 years. A total of 95.9% of them held a bachelor's degree or higher. The average team size was 3.95 (SD = 2.91).

### 3.2 Measures

We used a standard translation and back-translation procedure to remove bias caused by culture differences (Brislin, 1970). Unless otherwise indicated, all measures were based on a seven-point scale (1 = strongly disagree, 7 = strongly agree).

#### 3.2.1 Perceived leader competence (Time 1)

We measured perceived leader competence using a five-item scale developed by Fiske et al. (2002). A sample item was “My supervisor is competent” (α = 0.83).

#### 3.2.2 Employee hope (Time 2)

We measured employee hope using a six-item scale developed by Snyder et al. (1996). A sample item was “If I should find myself in a jam, I could think of many ways to get out of it” (α = 0.87).

#### 3.2.3 LMX (Time 2)

We measured LMX using a seven-item scale developed by (Graen and Uhl-Bien 1995). A sample item was “I usually know how satisfied my leader is with what I do” (α = 0.92).

#### 3.2.4 Self-improvement feedback-seeking (Time 3)

We measured self-improvement feedback-seeking using a five-item scale adapted from Janssen and Prins (2007). A sample item was “I ask my supervisor for feedback to learn how I can master tasks” (α = 0.94).

#### 3.2.5 Controls

Following the feedback-seeking behavior literature (Ashford et al., 2016), we controlled for participants' gender, age, education, and work tenure with leaders by regressing these variables to the mediating (i.e., employee hope) and dependent variables (i.e., self-improvement feedback-seeking behavior), as these demographic variables have potential effects on individuals' feedback-seeking behavior (Ashford et al., 2016). We also conducted the analyses without adding control variables, and the results showed the same significance.

### 3.3 Analytic strategy

To test the moderated mediation model, we applied Model 58 of the PROCESS macro in SPSS, using 5,000 bootstrap resamples to calculate bias-corrected confidence intervals. This analytical approach allowed us to simultaneously examine the mediating effect of hope and the contingent role of LMX at both stages of the mediation path. The variables involved in the interaction terms were mean-centered automatically by PROCESS to reduce multicollinearity and facilitate interpretation of the moderation effects. We analyzed our data at the individual level because the ICC (1) and ICC (2) values for our variables showed minimal between-group variance. Specifically, the ICC (1) and ICC (2) for perceived leader competence were 0.005 and 0.020, for LMX were 0.037 and 0.131, for hope were 0.020 and 0.073, and for self-improvement feedback-seeking were 0.043 and 0.151.

## 4 Results

### 4.1 Primary analyses

Before testing our hypotheses, we conducted a series of CFAs to confirm the distinctiveness of our focal variables. The results in Table 1 showed that the hypothesized four-factor model (i.e., leader competence, employee hope, LMX, and self-improvement feedback-seeking) fit the data well (χ^2^ = 1136.07, *df* = 224, *CFI* = 0.91, *TLI* = 0.90, *RMSEA* = 0.079, *SRMR* = 0.042) and was significantly better than the other alternative models based on χ^2^-difference tests, providing evidence for the discriminant validity of our focal variables.

**Table 1:** Model fit results for the confirmatory factor analyses.

**Models**		***χ^2^***	***df***	***χ^2^/df***	****Δχ* ^2^***	**CFI**	**TLI**	**RMSEA**	**SRMR**
4-factor	PLC, HO, LMX, SIF	1136.07	224	5.07	–	0.91	0.90	0.079	0.042
3-factor	PLC + LMX, HO, SIF	2182.82	227	9.62	1046.75^***^	0.81	0.79	0.114	0.095
	PLC + HO, LMX, SIF	2219.16	227	9.78	1083.09^***^	0.81	0.78	0.115	0.098
	PLC + SIF, HO, LMX	2259.12	227	9.95	1123.05^***^	0.80	0.78	0.116	0.108
	PLC, HO + LMX, SIF	2275.46	227	10.02	1139.39^***^	0.80	0.78	0.117	0.081
	PLC, HO + SIF, LMX	2906.32	227	12.80	1770.25^***^	0.74	0.71	0.134	0.151
	PLC, HO, LMX + SIF	3908.29	227	17.22	2772.22^***^	0.64	0.60	0.157	0.122

N = 660. PLC stands for perceived leader competence; HO stands for hope; LMX stands for leader member exchange; and SIF stands for self-improvement feedback; χ^2^ $=\chi_{3-factor}^{2}-\chi_{4-factor}^{2}$;

^***^p < 0.001.

The results in Table 2 showed that the vast majority of the factor loadings were close to and greater than 0.70 (except for two: HO1 and LMX1). Additionally, all of the average variance extracted (AVE) values exceeded the threshold of 0.50. Furthermore, the results in Table 3 indicated that the square roots of the AVE values were higher than the correlation coefficients between latent variables, satisfying the Fornell-Larcker criterion. Although not all factor loadings exceeded 0.70, when considering the CFA results, AVE values, CR values, and the Fornell-Larcker criterion, we concluded that the four-factor model was acceptable and that the distinctiveness of our variables was supported.

**Table 2:** Reliability and validity of studied constructs.

**Variables**	**Factor loadings**	**α**	**AVE**	**CR**
PLC1	0.763	0.834	0.504	0.835
PLC2	0.722			
PLC3	0.688			
PLC4	0.697			
PLC5	0.675			
HO1	0.586	0.867	0.542	0.875
HO2	0.763			
HO3	0.772			
HO4	0.627			
HO5	0.841			
HO6	0.793			
LMX1	0.591	0.923	0.638	0.924
LMX2	0.774			
LMX3	0.831			
LMX4	0.824			
LMX5	0.855			
LMX6	0.855			
LMX7	0.830			
SIF1	0.917	0.941	0.762	0.941
SIF2	0.930			
SIF3	0.889			
SIF4	0.889			
SIF5	0.725			

N = 660. PLC stands for perceived leader competence; HO stands for hope; LMX stands for leader member exchange; and SIF stands for self-improvement feedback. AVE, Average Variance Extracted; CR, Composite Reliability.

**Table 3:** Descriptive statistics and correlations among focal variables.

**Variables**	**Mean**	**SD**	**1**	**2**	**3**	**4**
1. Perceived leader competence (T1)	4.38	0.44	**(0.91)**	0.18^***^	0.24^***^	0.14^**^
2. Hope (T2)	4.84	0.98	0.17^***^	**(0.94)**	0.56^***^	0.29^***^
3. LMX (T2)	4.90	0.92	0.21^***^	0.51^***^	**(0.96)**	0.33^***^
4. Self-improvement feedback-seeking (T3)	4.77	1.17	0.13^***^	0.28^***^	0.32^***^	**(0.97)**

N = 660. T1/T2/T3 = Time 1/Time 2/Time 3. Square root of AVE is depicted in bold in parentheses along the diagonal. The correlation coefficients between latent variables are presented in the upper-right corner.

^***^p < 0.001, ^**^p < 0.01.

We also conducted a linear regression analysis incorporating all predictors—perceived leader competence, hope, LMX, and the two interaction terms—while assessing potential collinearity. The results showed that variance inflation factor (VIF) values for the five predictors ranged from 1.05 to 1.39 (lower than 10), with tolerance values falling between 0.72 and 0.95 (higher than 0.1; Spychala and Sonnentag, 2011). These indices confirm that multicollinearity was not a substantive concern in this study.

The descriptive statistics, correlations, and reliabilities of our focal variables are presented in Table 3. As predicted, leader competence was positively and significantly related to employee hope (*r* = 0.17, *p* < 0.001), and employee hope was positively and significantly related to self-improvement feedback-seeking (*r* = 0.28, *p* < 0.001), providing initial support for our hypotheses.

### 4.2 Common method variance tests

All of the variables in our study were single-rated, which may introduce the risk of for common method bias (CMB; Podsakoff et al., 2012). To handle this concern, we conduct Harman's one-factor analysis and the unmeasured latent method construct (ULMC) analysis (Podsakoff et al., 2003). The results indicated that the first factor accounted for 33.29% of the variance, which is below the threshold of 40% commonly used to indicate significant CMB. The ULMC test showed that adding the common method factor [χ^2^_(223)_ = 878.734, χ^2^/df = 3.94, CFI = 0.91, TLI = 0.89, RMSEA = 0.067, SRMR = 0.096] improved model fit compared to the four-factor model [χ^2^_(224)_ = 1136.07, χ^2^/df = 5.07, CFI = 0.91, TLI = 0.90, RMSEA = 0.079, SRMR = 0.042]. The significant Chi-square difference (χ^2^ = 257.336, *p* < 0.001) suggested a better fit, but several observations indicated that common method variance (CMV) was not a major concern in this study. First, while RMSEA improved, CFI and TLI remained largely unchanged, and SRMR worsened from 0.042 to 0.096, exceeding the critical threshold of 0.08. This suggested that the common method factor may not have been effectively capturing method variance, but instead was introducing specification error. Second, the minimal improvement in fit indices suggests that the better fit of the ULMC model is due to its added complexity, not a strong method factor. Thus, the common method factor did not account for significant variance in the core variables.

### 4.3 Hypothesis testing

Hypothesis 1 predicted a positive relationship between leader competence and self-improvement feedback-seeking. The results in Table 4 show that perceived leader competence was positively but not significantly associated with self-improvement feedback-seeking (*B* = 0.12, *SE* = 0.10, *n.s*., Model 2). Thus, Hypothesis 1 was not supported by the data. It was worth noting, however, that the zero-order association between perceived leader competence and self-improvement feedback-seeking was significant. The non-significant direct effect can be explained by the mediation of hope and the moderation of LMX, as proposed in Hypotheses 2–4.

**Table 4:** Results of the path analyses.

**Variables**	**Hope (T2)**		**Self-improvement feedback-seeking (T3)**	
	**Model 1**		**Model 2**	
	***B***	***SE***	***B***	***SE***
**Focal variables**				
Perceived leader competence (T1)	0.14^†^	0.08	0.12	0.10
LMX (T2)	0.54^***^	0.04	0.29^***^	0.06
Leader competence × LMX	0.21^**^	0.08		
Hope (T2)			0.18^***^	0.05
Hope × LMX			0.10^**^	0.04
**Controls**				
Gender (T1)	−0.12	0.12	0.16	0.16
Age (T1)	−0.02	0.02	−0.01	0.03
Education (T1)	0.11	0.09	0.03	0.11
Tenure with leader (T1)	0.02	0.02	0.01	0.03
***R* ^2^**	0.28		0.13	
***F*** **value**	*F*_(7647)_ = 35.99^***^		*F*_(8646)_ = 12.45^***^	

N = 660. T1/T2/T3 = Time 1/Time 2/Time 3.

^†^p < 0.1, ^**^p < 0.01, ^***^p < 0.001.

Hypothesis 2 predicted a positive relationship between leader competence and employee hope. The results in Table 4 show that leader competence was positively associated with employee hope (*B* = 0.14, *SE* = 0.08, *p* < 0.1, 95% CI [−0.017, 0.288], Model 1), indicating a marginally significant effect. Hypothesis 3 predicted the mediating effect of employee hope on the relationship between leader competence and self-improvement feedback-seeking. The results in Table 3 show that employee hope was positively associated with self-improvement feedback-seeking (*B* = 0.18, *SE* = 0.05, *p* < 0.001, 95% CI [0.080, 0.280], Model 2). In addition, the indirect effect of leader competence on self-improvement feedback-seeking via employee hope was 0.024 (*SE* = 0.02, *p* < 0.1, 95% CI [−0.004, 0.065]), indicating marginal significance.

**Figure 2:**
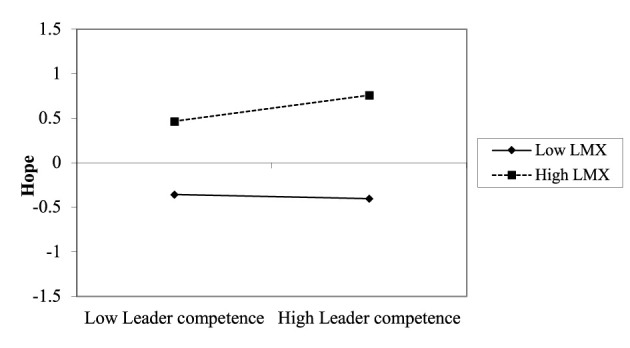
The interactive effect of leader competence and LMX on employee hope.

**Figure 3:**
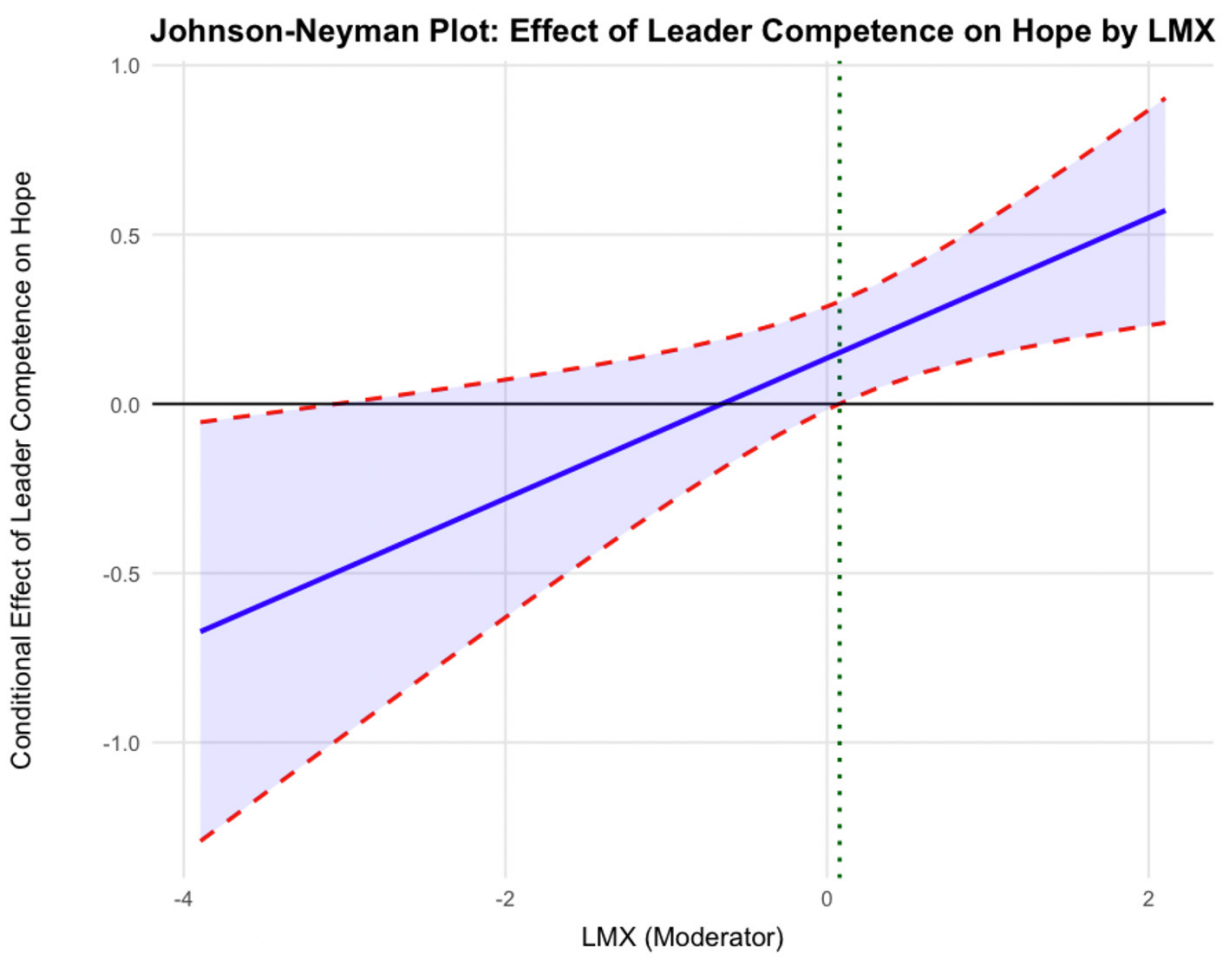
Johnson-Neyman plot illustrating the interactive effect of leader competence and LMX on employee hope.

Hypothesis 4a predicted the moderating role of LMX in the relationship between leader competence and employee hope. The results in Table 4 show that the interaction term (i.e., leader competence ^*^ LMX) was significantly associated with hope (*B* = 0.21, *SE* = 0.08, *p* < 0.05, 95% CI [0.058, 0.357], Model 1), with a change in explained variance of Δ*R*^2^ = 0.008, providing support for Hypothesis 4a. To further explain the moderating effect, we conducted simple slope tests. Figure 2 reveals that the relationship between leader competence and employee hope was positive and significant when LMX was high (*simple slope* = 0.33, *SE* = 0.10, *p* < 0.001), but this relationship was not significant when LMX was low (*simple slope* = −0.06, *SE* = 0.11, *n.s*.). Johnson-Neyman analysis (Figure 3) showed that perceived leader competence positively predicted hope when LMX was above 0.078, but not at lower levels, indicating that leader competence enhances hope only under sufficiently high-quality LMX. Thus, Hypothesis 4a was fully supported.

**Figure 4:**
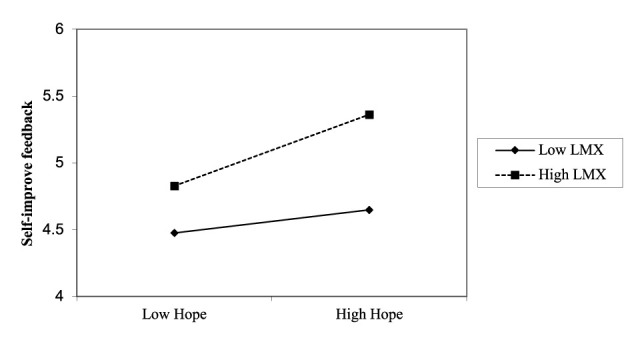
The interactive effect of employee hope and LMX on self-improvement feedback-seeking.

**Figure 5:**
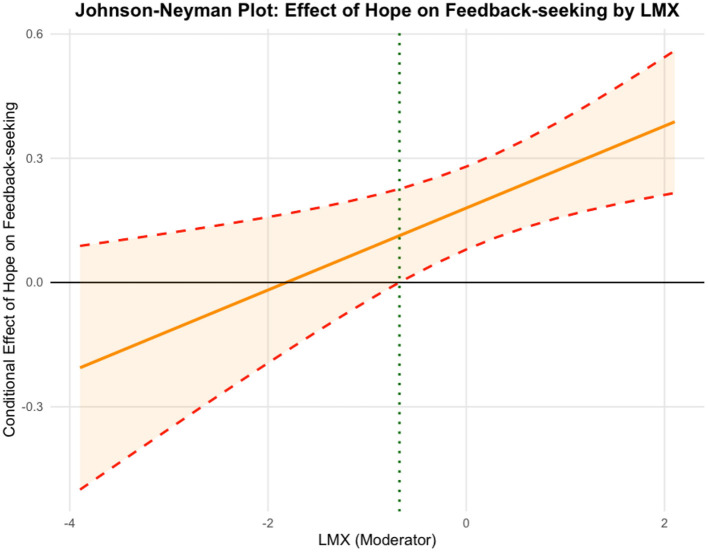
Johnson-Neyman plot illustrating the interactive effect of employee hope and LMX on self-improvement feedback-seeking.

Hypothesis 4b predicted the moderating role of LMX in the relationship between employee hope and self-improvement feedback-seeking. The results in Table 3 show that the interaction term (i.e., employee hope ^*^ LMX) was significantly associated with self-improvement feedback-seeking (*B* = 0.10, *SE* = 0.04, *p* < 0.05, 95% CI [0.0294, 0.169], Model 2), with a change in explained variance of Δ*R*^2^ = 0.011, providing support for Hypothesis 4b. Similarly, we conducted simple slope tests to further interpret the moderating role of LMX. Figure 4 reveals that the relationship between employee hope and self-improvement feedback-seeking was positive and significant when LMX was high (*simple slope* = 0.27, *SE* = 0.06, *p* < 0.001), but this relationship was not significant when LMX was low (*simple slope* = 0.09, *SE* = 0.06). Johnson-Neyman analysis (Figure 5) indicated that hope positively predicted self-improvement feedback-seeking when LMX exceeded −0.674, but not at lower levels, suggesting that hope promotes feedback-seeking mainly in the context of high-quality LMX. Thus, Hypothesis 4b was fully supported.

Although we did not hypothesize a moderated mediation, results from bias-corrected bootstrap analyses (5,000 resamples) showed that the indirect effect was significantly positive when LMX was high (*indirect effect* = 0.088, 95% Boot CI [0.005, 0.186]), but non-significant when LMX was low (*indirect effect* = −0.005, 95% Boot CI [−0.080, 0.025]). The index of moderated mediation was significantly different from zero (*index* = 0.037, *BootSE* = 0.018, 95% Boot CI [0.006, 0.076]), indicating that LMX significantly moderates the indirect effect of leader competence on self-improvement feedback-seeking through hope.

## 5 Discussion

Drawing from the self-improvement perspective, we examined how and when perceived leader competence impacts employees' self-improvement feedback-seeking. Through a time-lagged field study, we found that perceived leader competence has a marginally positive relationship with employees' self-improvement feedback-seeking through increasing their hope. In addition, LMX plays moderating roles in the relationship between perceived leader competence and employee hope as well as between employee hope and self-improvement feedback-seeking, with these two relationships becoming stronger when LMX is greater than lower.

### 5.1 Theoretical implications

Our study makes three theoretical contributions to the feedback-seeking literature and self-motives theory. First, we focus on the self-improvement perspective to the feedback-seeking behavior literature. Although the original intention of feedback-seeking was to attain a valued state (Ashford, 1986), most prior research has focused on the general component of employees' feedback-seeking, which means that individuals seek feedback about their overall performance, technical aspects of the job and so on Vandenberghe et al. (2021); VandeWalle et al. (2000). Little is known, however, about the self-improvement side of feedback-seeking. Moreover, although research has stressed the importance of self-improvement motives in feedback-seeking and developed measures for self-improvement feedback-seeking (Anseel et al., 2007; Janssen and Prins, 2007), little research has explored its activators and provided empirical evidence. In this research, we focused on employees' self-improvement feedback-seeking and found that perceived leader competence could trigger this behavior, which then enriched our understanding of the improvement nature of feedback-seeking behavior.

Second, we unveil the motivational mechanism (i.e., employee hope) of perceived leader competence and self-improvement feedback-seeking, through which we contribute to the hope literature by attaching hope to employee studies. Previous research has indicated that individual hope can generate positive outcomes such as improved academic and sports performance (Curry et al., 1997) and life satisfaction (Bailey et al., 2007); however, its function in the workplace has received less attention. Indeed, research has stressed the importance of hope in human resource development in the workplace (Luthans and Jensen, 2002; Reichard et al., 2013) and found that employee hope can generate higher levels of performance and work engagement (Lin et al., 2018; Ouweneel et al., 2012). We advance this research line by assuming hope as a form of individual self-improvement motive and proving its mediating role in the relationship between perceived leader competence and employees' self-improvement feedback-seeking.

Third, we also contribute to LMX theory by distinguishing the moderating effect of LMX in different contexts. With respect to perceived leader competence, prior research has paid little attention to the interactive effect of leader competence and leader-member relationship quality (with exceptions: Byun et al., 2017; Kacmar et al., 2007). However, these two studies primarily focus on the interactive effect of leader competence and LMX on performance or work effort (Kacmar et al., 2007). We advance this research line by considering both its effects on employee motivational state (i.e., hope) and found that the positive effect of perceived leader competence on hope is strengthened when LMX was high. When combined with employees' motivational states (i.e., hope) and employees' feedback-seeking, extant research has focused mainly on the intriguing effects of LMX on feedback-seeking behavior (Ashford et al., 2016; Lam et al., 2007). We advanced this by showing that the autonomy and fearlessness of failure accompanied by high LMX help transfer employee hope (i.e., motivational state) to self-improvement feedback-seeking.

It is important to note that the direct effect between perceived leader competence and self-improvement feedback-seeking was not significant, given the full mediation effect of hope under certain conditions. Moreover, the relationship between leader competence and hope, as well as the indirect effect on feedback-seeking via hope, were marginally significant at average levels of LMX. One possible explanation is that in high-reliability contexts, such as nuclear power, employees may need both psychological resources and relational safety (i.e., hope in the current study) before they can translate perceptions of leader competence into feedback-seeking behavior (e.g., Martínez-Córcoles et al., 2011). Therefore, the influence of perceived leader competence on feedback-seeking operates indirectly, through employee hope, and its strength depends on the quality of LMX.

### 5.2 Practical implications

Our study has several important practical implications. First, leader competence—even as perceived by followers—plays a crucial role in employees' self-improvement processes, including motivation and feedback-seeking behaviors. Leaders should therefore consciously cultivate a competent image and continuously develop their own leadership capabilities. Competency assessments can also be incorporated into selection or promotion processes to ensure leadership effectiveness (Levenson et al., 2006). Moreover, LMX serves as a cultural factor that plays an important role in fostering feedback-rich environments and enhancing performance (Mulia et al., 2021). Thus, while focusing on competence development, leaders should also pay attention to cultivating relational and cultural aspects.

Second, employee hope is identified as a key psychological resource that drives proactive learning behaviors. Organizations can enhance hope through interventions such as mindfulness training, fostering a growth mindset, or creating supportive work environments (Ouweneel et al., 2012). In the specific context of the nuclear power industry, we validate the roles of leader competence and LMX in motivating subordinates' psychological states and behaviors. This, to some extent, resonates with the emphasis on leadership agility in the VUCA era (Syamsir et al., 2025) and also offers insights into effective leadership during crises, such as the COVID-19 pandemic (Safitri et al., 2021).

### 5.3 Limitations and future directions

Our study also has some limitations that should be addressed by future research. First, we collected our data from one company located in China; thus, the issue of external validity exists. Moreover, although we measured variables at multiple time points, we did not include baseline self-improvement feedback at T1, which prevents us from examining changes in this behavior over time more accurately. Future research is encouraged to test the theoretical models with more diverse samples, incorporate ratings from multiple sources, and pay closer attention to behavioral changes over time.

Second, we primarily focused on one side of feedback-seeking behavior (i.e., the self-improvement side) in our study. Indeed, other types of feedback-seeking behavior, such as self-validation feedback-seeking and self-positive and negative feedback-seeking, are also worth considering (Gong et al., 2017; Janssen and Prins, 2007). Future research will benefit from considering these different types of feedback-seeking behavior simultaneously and exploring the discrepant effects of perceived leader competence on them.

Finally, we paid limited attention to the classification of leader competence. In detail, in the current study, we focused mainly on the emotional intellective side (Cuddy et al., 2011; Kong et al., 2023). However, leader competence consists of different components, such as emotional intelligence, which ensures smooth communication with subordinates (Wong and Law, 2017); competence targets one's own work; and employee mentoring (Kacmar et al., 2007; Levenson et al., 2006). In addition, we did not specifically consider leader warmth, which may jointly influence employee perceptions and behaviors alongside competence. Further research should thoroughly examine these different dimensions and their distinct functions.

## 6 Conclusion

Our research indicates that perceived leader competence can improve employees' self-improvement feedback-seeking behavior by increasing their hope (marginally). In addition, the relationship quality between leaders and employees (i.e., LMX) increases the positive effect of perceived leader competence on employee hope and that of employee hope on self-improvement feedback-seeking behavior. These results provide guidance for leadership development and employee self-improvement to inspire the precious positive emotional state which activate individual's proactive behavior.

## Data Availability

The raw data supporting the conclusions of this article will be made available by the authors, without undue reservation.

## Appendix

**Perceived leader competence** (Fiske et al., 2002)

1. My supervisor is competent (我的领导很有能力)
2. My supervisor is confident (我的领导很自信)
3. My supervisor is independent (我的领导很独立)
4. My supervisor is competitive (我的领导很有竞争力)
5. My supervisor is intelligent (我的领导很聪明)

**LMX** (Graen and Uhl-Bien, 1995)

1. I usually know how satisfied my leader is with what I do (我通常知道我的领导对我的工作有多满意)
2. My leader understands my job problems and needs well (我的领导很了解我工作中的问题和需求)
3. My leader recognizes my potential (我的领导认可我的潜力)
4. Regardless the formal authority my supervisor has, he/she would use his/her power to help me solve my problems at work (不管我的领导有多大的正式权力,他都会利用它帮我解决工作中的问题)
5. Regardless the formal authority my supervisor has, he/she would “bail me out” at his/her expense (不管我的领导有多大的正式权力,他都会在自己能力范围内为我解困)
6. I have enough confidence in my leader that I would defend and justify his/her decision if he/she were not present to do so (我对领导足够信任,即使他不在场,我也会为他的决策辩护)
7. In general, I have a good work relationship with my leader (总的来说,我与我的领导有良好的工作关系)

**Hope** (Snyder et al., 1996)

1. If I should find myself in a jam, I could think of many ways to get out of it (即使我陷入困境,我也可以想出很多办法摆脱它)
2. I energetically pursue my goals (我积极地追求自己的目标)
3. There are lots of ways around any problem (我能想出好多办法解决我面临的难题)
4. I see myself as being pretty successful (我觉得自己很成功)
5. I can think of many ways to reach my current goals (我能想出很多方法来实现我的目标)
6. I can meet the goals that I have ever set for myself (我能实现自己曾经定下的目标)

**Self-improvement feedback-seeking** (Janssen and Prins, 2007)

1. I ask my supervisor for feedback to learn how I can master tasks (我向领导寻求反馈以学习如何掌握任务要领)
2. I ask my supervisor for feedback to learn how I can improve performing my work (我向领导寻求反馈以学习如何提升工作绩效)
3. I ask my supervisor for feedback to get information about how I can solve problems (我向领导寻求反馈以收集有助于解决问题的信息)
4. I ask my supervisor for feedback to improve my knowledge and capabilities (我向领导寻求反馈以提升自己的知识与技能)
5. I ask my supervisor for feedback to set more appropriate goals myself (我向领导寻求反馈以设定更加合理的目标)
